# VO_2_max Trainability and High Intensity Interval Training in Humans: A Meta-Analysis

**DOI:** 10.1371/journal.pone.0073182

**Published:** 2013-09-16

**Authors:** Andrew P. Bacon, Rickey E. Carter, Eric A. Ogle, Michael J. Joyner

**Affiliations:** 1 Department of Anesthesiology, Mayo Clinic, Rochester, Minnesota, United States of America; 2 Department of Health Sciences Research, Division of Biomedical Statistics and Informatics, Mayo Clinic, Rochester, Minnesota, United States of America; 3 Creighton University Medical School, Omaha, Nebraska, United States of America; University of Bath, United Kingdom

## Abstract

Endurance exercise training studies frequently show modest changes in VO_2_max with training and very limited responses in some subjects. By contrast, studies using interval training (IT) or combined IT and continuous training (CT) have reported mean increases in VO_2_max of up to ∼1.0 L · min^−1^. This raises questions about the role of exercise intensity and the trainability of VO_2_max. To address this topic we analyzed IT and IT/CT studies published in English from 1965–2012. Inclusion criteria were: 1)≥3 healthy sedentary/recreationally active humans <45 yrs old, 2) training duration 6–13 weeks, 3) ≥3 days/week, 4) ≥10 minutes of high intensity work, 5) ≥1∶1 work/rest ratio, and 6) results reported as mean ± SD or SE, ranges of change, or individual data. Due to heterogeneity (I^2^ value of 70), statistical synthesis of the data used a random effects model. The summary statistic of interest was the change in VO_2_max. A total of 334 subjects (120 women) from 37 studies were identified. Participants were grouped into 40 distinct training groups, so the unit of analysis was 40 rather than 37. An increase in VO_2_max of 0.51 L ·min^−1^ (95% CI: 0.43 to 0.60 L · min^−1^) was observed. A subset of 9 studies, with 72 subjects, that featured longer intervals showed even larger (∼0.8–0.9 L · min^−1^) changes in VO_2_max with evidence of a marked response in all subjects. These results suggest that ideas about trainability and VO_2_max should be further evaluated with standardized IT or IT/CT training programs.

## Introduction

The benefits of an active lifestyle are well documented [1]–[3]. Many of these benefits are also associated with higher levels of cardiorespiratory fitness (VO_2_max) which may exert protective effects that are independent of traditional risk factors [3], [4]. Additionally, for individuals with low physical fitness, even modest improvements in fitness can have substantial health benefits. However, some individuals may have a limited ability to increase their cardiorespiratory fitness (trainability) in response to endurance exercise training [5], [6].

A key study advancing the idea that some humans have limited trainability comes from Bouchard et al. studied 483 sedentary white adults from 99 nuclear families who completed a standardized 20-wk endurance training program [5], [6]. The subjects trained three times per week on a treadmill. Initially, they trained at a heart rate that correlated to 55% of their baseline VO_2_max for 30 minutes per session. Every two weeks the intensity and duration of the exercise was progressively increased until each subject was training for 50 minutes at a heart rate associated with 75% of their baseline VO_2_max. This level of intensity and duration was reached by the 14th week of training and maintained until the conclusion of the study. Using this approach, they found a mean increase in VO_2_max of ∼0.4 L · min^−1^ with a SD of >0.2 L/min. Additionally, 7% of subjects showed a gain of 0.1 L · min^−1^ or less while 8% of subjects improved by 0.7 L · min^−1^ or more.

Based on this distribution of VO_2_max responses it appears that the “trainability” of at least some subjects is low or non-existent with little or no improvement in cardiorespiratory fitness in spite of 20 weeks of structured exercise training [5], [6]. These observations are in contrast to reports from smaller studies that have used either interval training (IT), or interval training in combination with continuous training (CT) and shown more robust increases in VO_2_max with at least some evidence of marked responses in all subjects [7]–[15].

In this context, we sought to explore the hypothesis that all subjects can show marked improvements in VO_2_max if training programs that include periods of high intensity (∼90% of VO_2_max) exercise are used. A fundamental rationale underpinning our analysis is that the biology of VO2max trainability needs to be evaluated using regimens designed to maximize physiological adaptations. To test this hypothesis, we evaluated the changes in VO_2_max in response to interval training (IT) or combined IT and continuous training (CT) reported in 37 studies [7]–[43]. We also sought to gain insight into the idea that shorter periods of IT might be either superior or more time efficient at generating increases in VO2max in comparison to traditional continuous training.

## Methods

### Study Eligibility Criteria and Literature Search

**Figure 1:**
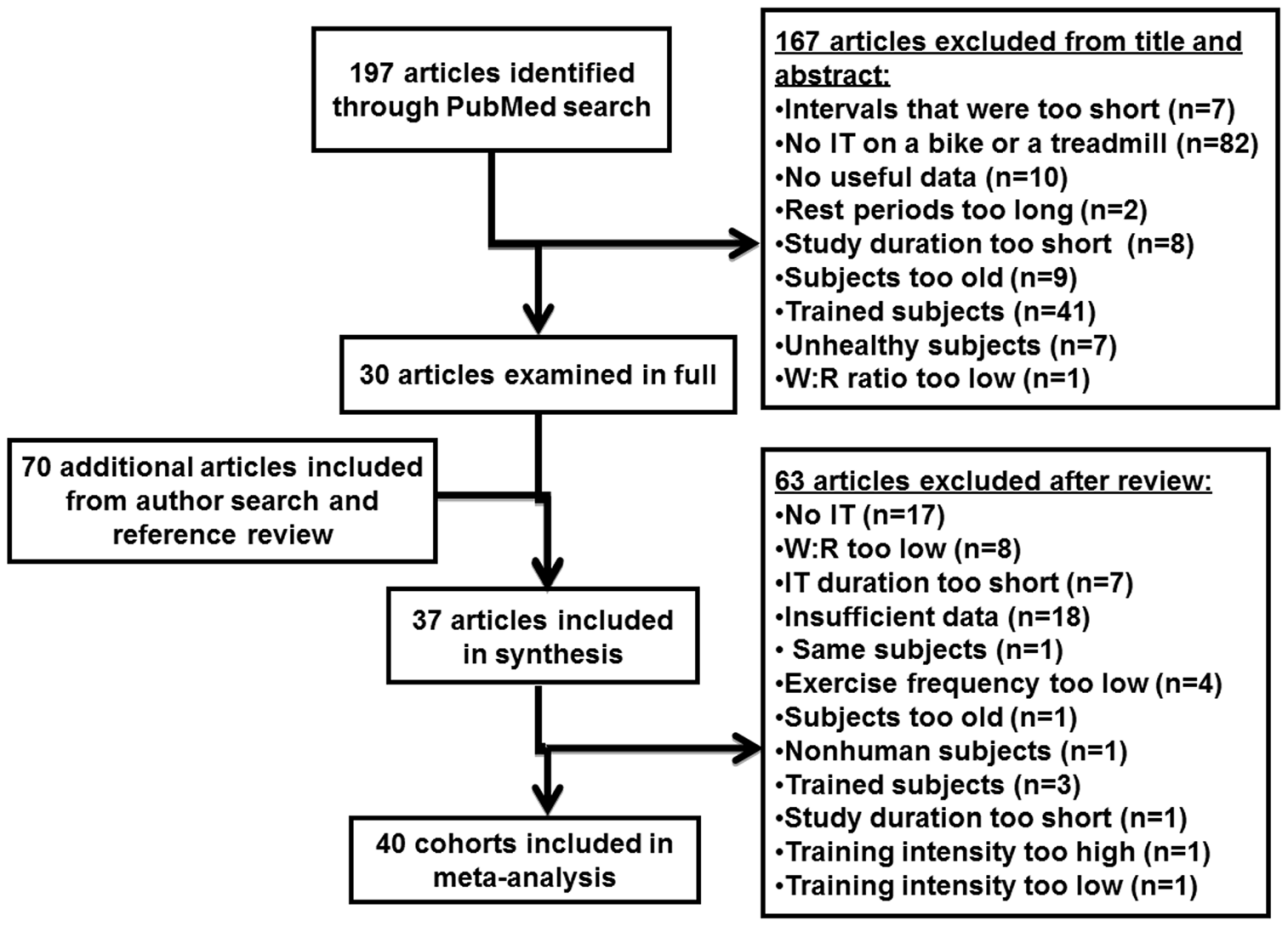
Schematic of our study identification and screening approach.

A search of PubMed was developed and conducted by a reference librarian looking for English language studies occurring between the years of 1968–2010 using the following search terms: interval training, VO_2_max, interval exercise + high/low intensity, maximum O_2_, maximum/maximal VO_2_, maximal oxygen consumption, peak oxygen uptake, maximal aerobic capacity, and the following limits: humans, English. The primary author of each eligible study was searched for in PubMed using the authors name and the search term: exercise. Searches were also run on other authors known by the investigators to have published papers that used a combination of interval and continuous training. The references of all eligible studies were also reviewed to identify other potentially eligible studies that might have been missed using the approaches outlined above. Subsequently, these studies were searched for in PubMed using the specific authors and article reference titles. Only published material was used. Figure 1 is a schematic of our search strategy.

In order to be considered eligible for inclusion, studies had to meet the following criteria:

1. Studies had to include a minimum of 3 subjects.
2. All subjects were previously healthy and between 18–45 years of age.
3. The initial fitness level of the subjects had to fall within the range of untrained/sedentary to recreationally active, defined as 55 ml · kg^−1^ · min^−1^ or lower for men and 49.5 ml · kg^−1^ · min^−1^ or lower for women. Studies were included with recreationally trained individuals if they were in the minority and if individual data was reported, allowing us to recalculate the pre and post training VO_2_max values and their associated SDs without the trained individuals. When VO_2_max was not provided in ml · kg^−1^ · min^−1^ a group estimate was calculated using the mean weight and pre VO_2_max in L · min^−1^.
4. Subjects had to train a minimum of 3 days per week for 6–13 weeks. At least two, and preferably three, training sessions had to be interval training.
5. Subjects had to be trained on either a cycle ergometer or by running with a minimum work: relief ratio of 1 min on:1 min off.
6. During each interval training session subjects had to have a minimum cumulative time of 10 minutes at high intensity. Due to the heterogeneity of training programs in the various studies we felt it was critical for the “work” period of the exercise sessions to be at intensities that were likely at least 80–85% of VO2max or higher. In some studies the desired intensities were set and monitored rigorously via power output or heart rate and in some cases the authors had to make a reasoned estimate. This was especially the case with some of the running training studies that used timed runs (for example reference 23).
7. Results had to be reported in pre and post mean VO_2_max values ± SD or SE, as ranges of change, or individual data.
8. Subjects could not be engaged in high intensity training before the start of the study.

### Study Selection

Two independent investigators (M.J.J and A.B.) screened the titles and abstracts of all studies identified by the various search methodologies to determine their potential eligibility. Studies that did not have an abstract, along with studies that were deemed potentially eligible, had their full text reviewed in order to determine if they met the criteria for inclusion in the meta-analysis. In order to avoid double counting of studies that reported different aspects of the physiological responses to training, the study design (training methodology, number of subjects, study duration) and subject characteristics (age, pre and post VO_2_max) of all accepted studies were compared. When studies with common authors, study design, and subject characteristics were found, the study with the larger number of subjects was used. This was done because it appeared that some training studies generated multiple publications with, for example, cardiorespiratory data the focus of one paper, and the metabolic adaptations to training the focus of a second paper.

### Data Extraction and Synthesis

Data extraction was performed by one investigator (A.B.) who used a standardized form that allowed for the extraction of study characteristics (author, title, number of subjects, study duration, age range, work: relief ratio, frequency of training, mean pre and post training VO_2_max values in L · min^−1^ and in ml · kg^−1^ · min^−1^ along with the associated standard errors of the mean (SEM) and standard deviations (SD)).

When needed, pre and post VO_2_max values, along with pre and post SD values, were converted into L • min^−1^ to limit the potentially confounding effects of changes in body weight that might have occurred with training. Pre and post SEM values were converted into SD. The calculated values were included in the data extraction sheet. For several studies mean, SD, and pre post correlation were recalculated from individual data. In the majority of studies, only the pre and post measurements were reported (i.e., the standard deviation of the change or the correlation of the pre and post measurements were not reported). Accordingly, the correlation was conservatively set at 0.5 for these cases. When studies reported treadmill and cycle VO_2_max values, the treadmill values were used.

### Summary measures

The principle summary measure utilized was group difference in pre and post training means for VO_2_max expressed in L • min^−1^, calculated with a 95% confidence interval using a random effects model [44]. As a sensitivity analysis to the random effects model, a fixed effects model was also fit to the data to estimate the degree of heterogeneity, indicated by I^2^. We used L • min^−1^ to avoid the potential confounding effect of significant weight loss and to better compare our analysis with the data from Bouchard et al. [5]. Mean difference was employed as it allowed us to calculate the effect size, which was then compared to the effect size of Bouchard et al. [5]. A standardized change in VO_2_max (mean change for each study divided by the standard deviation of change) was also analyzed using the same random effects modeling framework.

**Figure 2:**
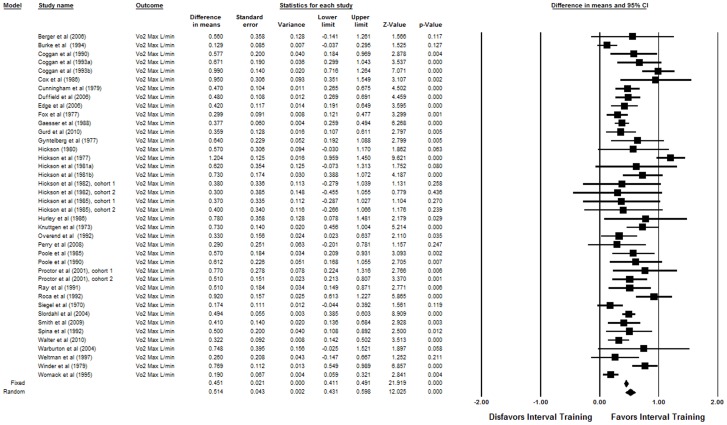
Funnel plot of standard errors observed for the reported articles by the reported difference in means. The open circles are observed studies whereas the solid circles are imputed studies to assess the role of publication on the estimated effect. The two estimates, denoted by the open and filled diamonds for the observed and imputed studies, respectively, agree qualitatively with one another suggesting that the findings may be robust in the presence of publication bias.

In order to evaluate the possibility of publication bias, a funnel plot of the mean differences was constructed and assessed. The funnel plot (Figure 2) indicated the potential for publication bias (e.g., smaller studies had larger effect sizes reported), so an imputed model was considered with fictional studies representing the complementary effect sizes to observed studies. A ‘Trim and Fill’ analysis was conducted to further assess for publication bias by providing an estimated pooled effect with the imputed studies while ‘trimming’ (removing) the most extreme small studies [45]. Comprehensive Meta Analysis Software (Biostat) version 2.2.064 was used for all analyses.

## Results

### Study Characteristics

A total of 211 articles were identified in the initial search. After screening titles and abstracts, 181 articles were discarded as it was apparent that they did not meet the study criteria. Figure 1 is a schematic of our search strategy and displays the criteria for exclusion. The full text of the remaining 30 articles was examined in detail. An additional 70 articles were identified through reference searches. The systematic review resulted in 37 articles for the Meta-analysis. Within these 37 articles were 334 participants (120 women) and 40 unique training groups (i.e., three articles each contained the results of two training programs). In brief, all studies contained between 3 to 19 healthy subjects with an age range of 18 to 42 years and pre training VO_2_max values ranging from ∼26 to 52 ml · kg^−1^ · min^−1^.

All subjects trained between 6 and 12 weeks using running, cycling, or a combination of the two modalities with a work relief ratio between 1∶1 and 5∶2. In all but six studies [10], [14], [15], [17], [32], [38] it was readily apparent that the subjects had trained at high intensity for 10 minutes or more. Knuttgen and Overend reported their work: relief ratios and the duration of their interval training, which allowed us to estimate the number of intervals performed and the time spent performing high intensity exercise which was determined to be greater than 10 minutes [15], [32]. Slordahl reported the duration of their interval training but reported their work: relief ratios as a range [38]. This allowed us to roughly estimate that the subjects performed at least 10 minutes of work at high intensity. Burke reported that their subjects trained to exhaustion; therefore it was assumed that they performed at least 10 minutes of work at high intensity [17. Warburton reported that their subjects performed interval training until the amount of work performed equaled the amount of work that would have been performed by the subject if they had been performing continuous training [14]. Since the subjects in the continuous training group worked for 30–48 min/day at 64.3±3.7% of their VO_2_max we assumed that the subjects performing interval training worked for at least 10 minutes at high intensity. Finally, Roca reported that their subjects trained less than 10 minutes at high intensity on one day of their high intensity training. Additionally, this was the only study in which interval training did not account for at least half of all training days for the test subjects. However, this exception was justified because the continuous component was judged to be strenuous enough to make up for the diminished interval component [10].

### Physiological Responses: Meta-Analysis Results

As a result of the training programs, VO_2_max increased (p<0.001). The estimated mean change in VO_2_max over the training programs using the random effects model was 0.51 L · min^−1^ (95% CI: 0.43 to 0.60 L · min^−1^) (Figure 2). In terms of standardized effect sizes (estimate divided by standard deviation), the change in VO_2_max was 0.86 SDs (95% CI: 0.72 to 0.99 SDs).

There was some indication of publication bias in the funnel plot (Figure 2). Specifically, the estimated effect using the ‘Trim and Fill’ approach was 0.37 L · min^−1^ (95% CI: 0.28 to 0.46 L · min^−1^), but the number of negative studies required to overturn the observed finding was estimated to be over 4000 (Rosenthal's fail-safe N = 4531) [46]. Thus, while Figure 2 shows a slight attenuation of the result with hypothetical studies imputed to account for potential publication bias, the results are not meaningfully changed. Finally, a sensitivity analysis was also conducted under a fixed effects model. There was significant heterogeneity in the exercise groups (I^2^ = 70), but the results of this analysis agreed qualitatively with the primary random effects model. Specifically, under the fixed effects model, the estimated change was 0.45 L · min^−1^ (95% CI: 0.41 to 0.49 L · min^−1^; p<0.001).

**Figure 3:**
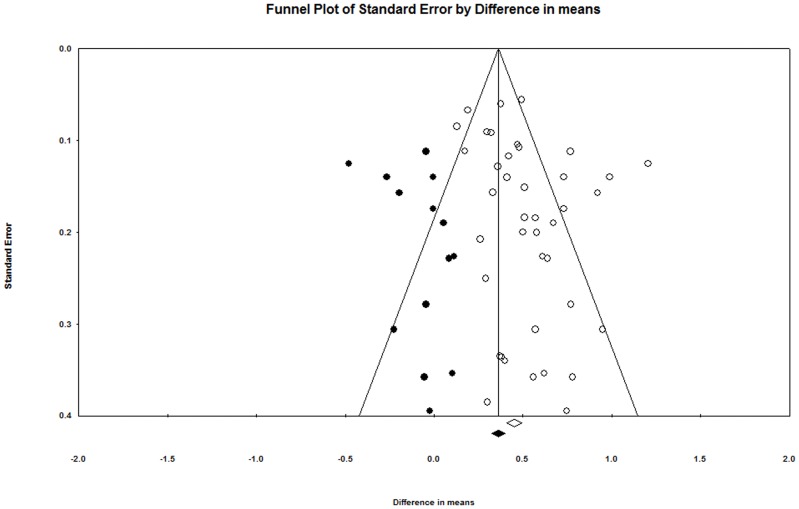
Forest plot for the synthesis of 37 articles (40 trained cohorts) identified in the systematic review. The estimates reported for each study are the means and 95% confidence intervals for the change in VO_2_max in L · min^−1^. The summary diamonds at the bottom of the plot represent the summarized effects using fixed and random effects models, where the random effects estimates are considered the primary findings for this study due to heterogeneity (I^2^ = 70). The estimated increase in VO_2_max as a result of interval training was 0.51 L · min^−1^ (95% CI: 0.43 to 0.60 L · min^−1^; p<0.001).

**Figure 4:**
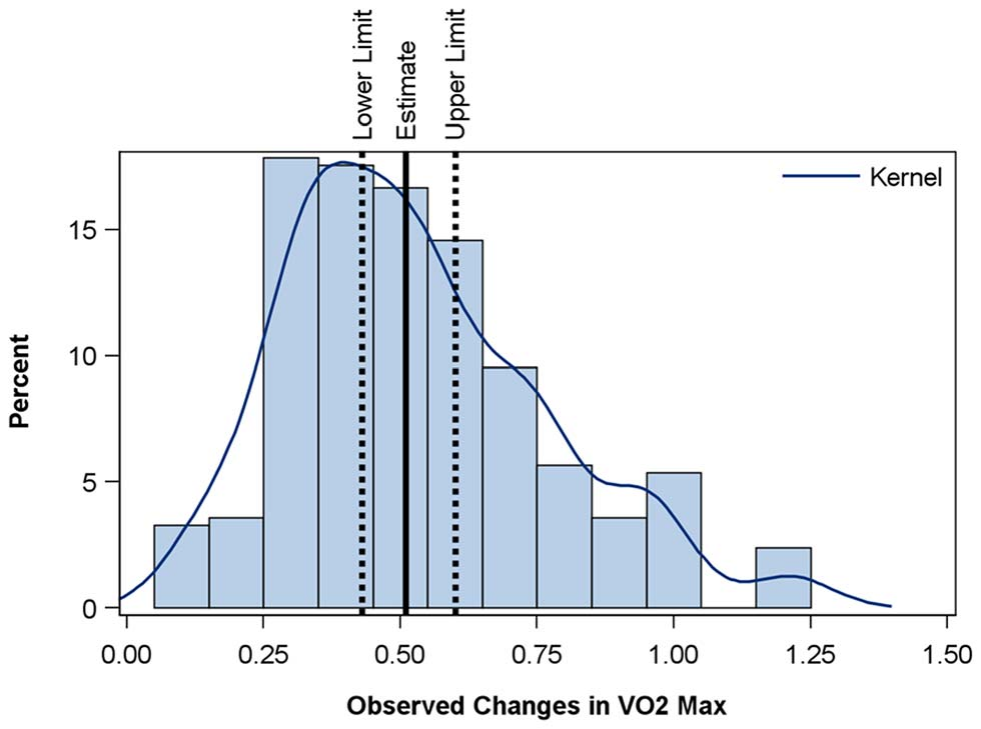
Weighted histogram and density plot of the observed effects of interval training on VO_2_max. Each of the 40 observations included in the analysis was weighted by the sample size to produce the distribution with the estimate of the percent of subjects with a given effect size noted on the Y axis.

To better illustrate the distribution of effects observed through the systematic review and demonstrated in Figure 3, a weighted histogram and density plot of the observed effects was created (Figure 4). Each of the 40 observations included in the analysis was weighted by the sample size to produce the distribution.

In a supplemental analysis, we also evaluated the nine studies that reported the largest [7]–[15] and nine studies that reported the smallest [17], [23], [30], [32], [33], [37], [41]–[43] mean changes in VO_2_max. These studies are the ones that would be located in the tails of the empirical distribution. Specifically, the mean ± SD increase in VO_2_max was 0.27±0.05 vs 0.87±0.15 L · min^−1^ (p<0.001) in this comparison. Weeks of training (6.9±1.4 vs 9.7±1.8; p = 0.004), duration per week (123±67 vs 209±90 min; p = 0.06), and the duration of the intervals for the entire study (479±246 vs 696±264 min; p = 0.12) showed general patterns that suggested the nine studies showing the smallest increases in VO_2_max were of shorter duration in terms of both weekly training and number of weeks. They also appear to have been less intense (fewer total high intensity minutes) than the nine studies showing the largest increases, although statistical significance was not achieved for all comparisons. Finally, many of the interval training studies reporting large increases in VO_2_max also used longer (3–5 min) duration intervals.

## Discussion

The main finding of this meta-analysis is that interval training produces improvements in VO_2_max slightly greater than those typically reported with what might be described as adult fitness based continuous training even though many of the studies were of short duration with limited training sessions per week. While the observation that more intense training results in greater increases in cardiorespiratory fitness is not surprising, our analysis suggests that longer intervals combined with high intensity continuous training can generate marked increases in VO_2_max in almost all relatively young adults.

As noted in the introduction, the Heritage Study demonstrated a mean increase in VO_2_max of 0.4 L · min^−1^ in response to 20 weeks of standardized adult fitness style training and marked individual variation was observed [5]. This value is also relatively high in comparison to other large adult fitness style training programs that show increases in VO_2_max on the order of 0.2–0.3 L • min^−1^ [47]. By contrast, the studies we evaluated that used interval training alone or in combination with continuous training estimated a mean increase in VO_2_max of 0.5 L · min^−1^. Figure 4 demonstrates an estimated distribution of training evoked changes in VO_2_max for the studies we evaluated. This figure presents a distribution of means whereas the Heritage data is of population values. It is worth noting, however, that the distribution of study effects presented in Figure 4 would appear to be shifted right in comparison to the Heritage data in spite of the fact that some of the studies were of limited duration (6–8 weeks); used intervals that were only one minute long and the time spent during the “work” portion of the interval training session was as low as 10 minutes. Thus, assuming the individual subject data used to generate the summary statistics in this meta-analysis follow roughly a normal distribution, the distribution of individual changes would be comparable, if not slightly better, than the Heritage data even though the overall duration (weeks spent training) were shorter in the studies we evaluated.

The conventional wisdom is that intervals of 3–5 minutes are especially effective in evoking increases in exercise capacity. Consistent with this idea, the nine studies that generated the biggest increases in VO_2_max (∼0.85/min) generally used intervals of 3–5 minutes and high intensity continuous training [7]–[15]. Additionally, many of these studies presented either individual data or ranges for VO_2_max values pre and post training, and inspection of this data suggests that a marked training response was seen in all subjects. Thus, given the large increases in VO_2_max seen in these studies, we believe that caution must be used in concluding that at least some humans are incapable (perhaps for genetic reasons) of increasing their VO2max in response to endurance exercise training.

Along these lines, many of the studies showing the largest increases in VO_2_max appeared to follow a pattern similar to the so-called “Hickson protocol” [7]. This protocol includes 10 weeks of training 6 days/week with interval and continuous training on alternate days. Interval training consists of six 5 minute sessions on a cycle ergometer at a work rate approaching the subjects' VO_2_max. These work periods are separated by 2 minutes of active rest. As the subjects' power output increases during training, the exercise intensity is increased as needed. On the non-interval days, continuous training consists of running as fast as possible for 30 min/day during the 1st week, 35 min/day during the second week, and 40 min/day or longer thereafter.

The original study of Hickson and colleagues noted that VO_2_max was continuing to increase at the end of the 10 week training program [7]. However, their subjects declined to continue to train due to the extremely arduous nature of the program. This observation emphasizes that it may be unrealistic to expect significant segments of the population to participate in an exercise training program that permits them to reach their individual upper limit for VO_2_max. It should also be noted that the high intensity continuous running almost certainly contributed to the large increases in VO_2_max seen in studies using the Hickson protocol but it is not possible to determine the relative role of each type of training.

Since there is no genetic data on the subjects from the studies included in our analysis it is not possible to use them to retrospectively evaluate the genetic determinants of trainability that have emerged from Heritage [5]. It is well accepted that the increases in VO_2_max with training are due to increases in cardiac output and peripheral oxygen extraction. However, the contribution of changes in stroke volume, blood volume, capillary density, muscle mitochondrial content and many other factors associated with training induced increases in VO_2_max might vary on both an individual basis and perhaps via interaction with specific elements of a given training program [48]–[52].

In addition to the well-known limitations associated with retrospective analysis of data reported previously, there are specific limitations to our analysis. First, as detailed above there was wide variability in training programs used in the studies we evaluated. Second, about two thirds of the subjects included in the studies we evaluated were (likely Caucasian) young men and this could explain at least some of the increased responsiveness that we saw. However, sex and age generally do not have major effects on the VO_2_max responses to training [5], [41]. We also did not include studies in patient populations that have shown marked improvements in exercise capacity with interval training in groups previously thought to have limited trainability [53]. Nor did we include a body of work showing that very short bouts of high intensity exercise followed by longer periods of recovery can have profound effects on exercise capacity [54]. The findings from these studies along with the data reported in our paper indicate that some of the peripheral metabolic adaptations to training require can be elicited by very short periods of high intensity exercise, but that longer intervals are required to see large changes in cardiac output and VO_2_max. The analysis also suggested some effect of publication bias, particularly with studies with small sample sizes and small effects. While the estimated change in VO_2_max remained statistically significant with various sensitivity analyses, a clear causal association is inherently limited with this data.

In summary, our analysis indicates that in addition to studies using training programs consistent with various public health guidelines, the basic biology of trainability needs to be evaluated using regimens designed to generate the most robust possible physiological adaptations.

## Supporting Information

Checklist S1 **PRISMA Checklist.**
(DOC)

Flowchart S1 **PRISMA Flowchart.**
(DOC)
