# Supplementary material for: VO2max Trainability and High Intensity Interval Training in Humans: A Meta-Analysis
Source: PLoS One. 2013 Sep 16;8(9):e73182. doi: 10.1371/journal.pone.0073182 (PMC3774727; doi:10.1371/journal.pone.0073182)
Supplement: Flowchart S1 — PRISMA Flowchart. (DOC) [file pone.0073182.s002.doc]

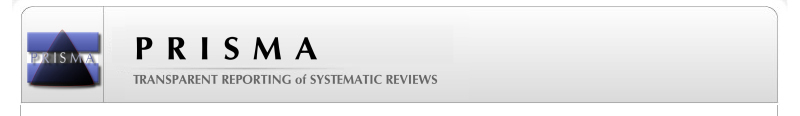
**PRISMA 2009 Flow Diagram**

Vc c

**Screening**

**Included**

**Eligibility**

**Identification**

Records identified through database searching
(n = 197)

Additional records identified through author search and reference search
(n = 1093)

Records after duplicates removed
(n = 1184)

Records screened
(n = 1184)

Records excluded
(n = 1084)

Full-text articles assessed for eligibility
(n = 100)

Full-text articles excluded:
(n = 63 )

No IT (n=17)

W:R too low (n=8)

Insufficient data (n=18)

Same subjects (n=1)

Exercise frequency too low (n=4)

Subjects too old (n=1)

Nonhuman subjects (n=1)

Trained subjects (n=3)

IT duration too short (n=7)

Study duration too short (n=1)

Training intensity too high (n=1)

Training intensity too low (n=1)

Studies included in qualitative synthesis
(n = 37)

Studies included in quantitative synthesis (meta-analysis)
(n = 37)
